# Perceived Outcomes, Parental Satisfaction, and Oral Health-Related Quality of Life After Full Mouth Rehabilitation Under General Anesthesia for Early Childhood Caries

**DOI:** 10.7759/cureus.47126

**Published:** 2023-10-16

**Authors:** Mebin George Mathew, Ganesh Jeevanandan

**Affiliations:** 1 Pediatric and Preventive Dentistry, Saveetha Dental College and Hospitals, Chennai, IND

**Keywords:** full mouth rebilitation, perceived outcomes, parental satisfaction, oral health-related quality of life, general anesthesia, early childhood caries

## Abstract

Aim: The aim of this article is to evaluate the perceived outcomes, parental satisfaction, and oral health-related quality of life after full mouth rehabilitation under general anesthesia for children with early childhood caries.

Materials and methods: A total of 200 children requiring full-mouth rehabilitation under general anesthesia for the management of early childhood caries were recruited for the study. Perceived outcomes, oral health-related quality of life, and parental satisfaction were evaluated at the follow-up visit after six months.

Results: All participants reported back for the follow-up visit after six months. Oral health-related quality of life after full-mouth rehabilitation showed statistically significant improvement at follow-up (P <0.001); 97.5% of the parents were satisfied with the treatment. Perceived outcomes were found to be satisfactory, and a significant improvement (P <0.001) was seen after treatment.

Conclusion: The perceived outcomes of participating parents were met. Significant improvement in oral health was seen after full-mouth rehabilitation under general anesthesia. Parental satisfaction was found to be high at the follow-up visit after six months. Parents found the improvement in the oral and general health of their child to be significant.

## Introduction

Early childhood caries (ECC) is the occurrence of a cavitated or non-cavitated, missing, or decayed tooth in children younger than 71 months of age [1]. ECC has debilitating effects on the general health, oral health, academic performance, financial burden, and quality of life of children as well as their families [1,2].

Children with ECC are very young and usually lack the cooperative ability required for dental treatment in the dental operatory. Because ECC spreads quickly to involve the teeth, treatment needs are usually high, and children also require invasive treatment such as pulp therapy or extraction [3]. To achieve the best possible outcomes, these children are often treated under general anesthesia. Comprehensive dental treatment is completed in a single appointment, and the child is discharged on the same day [4]. Though often considered the last resort for the management of ECC, the procedure should result in improvement and outweigh any risks associated with general anesthesia [3,5].

Parental satisfaction with full-mouth rehabilitation under general anesthesia has been found to be very high [3,5,6]. Opinions from parents help in improving the provision of general anesthesia. ECC affects not only the child but also the family. Parents must take time off from work, and management is usually a financial burden for families [5-7]. 

The outcomes for children who receive full-mouth rehabilitation under general anesthesia have been shown to depend on both the type of materials used and the type of procedures performed [7,8]. The management of children with ECC under general anesthesia has been studied for a long time, but parental satisfaction and perceived outcomes have rarely been explored [7-9]. This study was undertaken to evaluate oral health-related quality of life, perceived outcomes, and parental satisfaction after full-mouth rehabilitation under general anesthesia for ECC.

## Materials and methods

This prospective interventional cohort study was initiated after approval was obtained from the Institutional Human Ethical Committee and Scientific Review Board of the Saveetha Dental College and Hospitals, Saveetha Institute of Medical and Technical Sciences, Chennai, India (approval number: IHEC/SDC/FACULTY/21/PEDO/2810).

Children aged between three and six years who were diagnosed with ECC and required comprehensive dental treatment under general anesthesia were included in the study. Children with special healthcare needs, those who did not belong to the age group, and those who could be managed in the dental office were not included in the study. A total of 621 children diagnosed with ECC were seen in the Department of Pediatric and Preventive Dentistry of Saveetha Dental College and Hospitals, out of which 200 were found eligible based on the selection criteria and consented to the study. Parents were informed about the project in detail in a language they understood and in which they could converse well. All parents were given opportunities to clarify their doubts with the pediatric dentist and the anesthesiologist about the procedure and postoperative care.

The operating team followed a standardized treatment protocol for all patients. Carious lesions affecting the occlusal surface were restored with glass ionomer cement or composite. Stainless steel crowns were used to rehabilitate primary molar teeth that had carious lesions involving multiple surfaces whereas strip crowns were placed for anterior teeth with multisurface lesions. Teeth found to have pulpal involvement were treated with pulp therapy based on the guidelines of the American Academy of Pediatric Dentistry, followed by the placement of the final restoration; anterior teeth were restored with strip crowns and posterior teeth with stainless steel crowns. Teeth with necrotic pulps and non-restorable teeth were extracted under local anesthesia. Space maintainers were placed after extraction based on the clinical scenario. Preoperative and postoperative photographs were taken and uploaded into the dental information archival system of Saveetha Dental College and Hospitals, Saveetha Institute of Medical and Technical Sciences, Chennai.

On the day of the initial examination, preoperative interviews were performed and the chief complaints and dental history were recorded. Following the recovery period and before discharge, parents received a questionnaire for oral health-related quality of life, parental satisfaction, and perceived outcomes. This was to ensure their readiness for a postoperative interview during the next visit. Children were recalled after a week for an early follow-up. To evaluate parental adherence to the postoperative care plan, oral health-related quality of life, parental satisfaction, and perceived outcomes, postoperative interviews and clinical checkups were conducted six months after treatment completion.

Data collection

Oral Health-Related Quality of Life

To assess oral health-related quality of life in the present study, the Early Childhood Oral Health Impact Scale (ECOHIS) was used [10]. This is a validated and reliable tool to measure the oral health-related quality of life of preschool children. ECOHIS consists of two sections (Child Impact Section and Family Impact Section) and 13 questions for the parents to answer.

Perceived Outcomes and Parental Satisfaction

Perceived outcomes and parental satisfaction were collected based on the survey used by El Batawi et al. [9]. Oral symptoms, functional limitations, emotional satisfaction, and social well-being were the domains explored in perceived outcomes. Parents gave a yes or no answer to all domains before and after the treatment. Parental satisfaction was evaluated at the end of the follow-up based on the parents’ experience and if they were willing to consider general anesthesia for full-mouth rehabilitation for their children again (Table 1).

**Table 1:** Perceived outcome questionnaire filled by parent or caregiver accompanying the child

Questions
I. Oral symptoms
a. Food caught between teeth
b. Pain in teeth/mouth
c. Bad breath
d. Mouth sores
e. Bleeding gums
II. Functional limitations
a. Difficulty chewing coarse food
b. Difficulty drinking or eating hot/cold foods
c. Difficulty eating foods that the child would like to eat
d. Restricted diet
e. Troubled sleeping
III. Emotional and social well being
a. Worried of being less attractive
b. Shy/embarrassed
c. Avoided smiling in front of strangers
d. Did not want to speak or read in class
e. Asked by other children about the condition
f. Missed school
IV. Parental satisfaction
a. Overall experience is satisfactory
b. Would consider general anesthesia for treatment again
c. In case of available safe sedation, would you prefer multiple visits over one‑visit general anesthesia?

Statistical analysis

Clinical and questionnaire data were entered into Excel (2019; Microsoft Corporation, Redmond, Washington, United States) and analyzed using IBM SPSS Statistics for Windows, Version 23.0 (Released 2015; IBM Inc, Armonk, New York, United States). Data analysis included descriptive statistics and bivariate tests. p-value ≤0.05 was considered significant.

## Results

In the present study, 104 participants (52%) were males, and the remaining 96 participants (48%) were females. Of the children, 70% were accompanied by their mothers and the remaining 30% were accompanied by their fathers. The mean age of the participants was 4.09 ±1.62 years. The participants’ mean decayed, missing, and filled primary teeth (DMFT) score was found to be 12.79 ± 4.32. The demographic data of the participants is summarized in Table 2.

**Table 2:** Demographic details of the participants SD: standard deviation; DMFT: decayed, missing, and filled primary teeth

Parent demographics	N (%)
Relationship to the child	
Mother	140 (70)
Father	60 (30)
Education level	
Secondary or below	154 (77)
Post-secondary or above	46 (23)
Child Demographics	
Gender	
Male	104 (52)
Female	96 (48)
*Age* (years), mean±SD	4.09±1.62
DMFT score, mean±SD	12.79±4.32

Of the participants, 99% required pulp therapy for primary teeth, with 8.02±1.79 teeth treated on average; 99% required restorations, with an average of 8.93±1.64 restored per participant. Stainless steel crowns were required by 96% of the participants, 13% required extractions, and 14% of the participants required space maintainers. Table 3 gives the details of the treatment done for the participants under general anesthesia.

**Table 3:** Dental treatment provided in the study for the participants under general anesthesia SD: standard deviation

Procedure	Children who received the treatment, n (%)	Average treated tooth, mean±SD
Pulp therapy	198 (99)	8.02±1.79
Restorations	198 (99)	8.93±1.64
Stainless steel crowns	192 (96)	2.13±0.71
Extractions	26 (13)	3.61±1.12
Space maintainer	28 (14)	3.04±1.26

Perceived outcomes by the participants’ parents were found to be significantly high. All three domains showed statistically significant improvement (P<0.001). Pain was the most common oral symptom (95.5%) before treatment and was found to have decreased significantly after intervention. All five components of oral symptoms showed statistically significant improvement after treatment (P<0.001). Eighty-five (42.5%) participants had difficulty in sleeping before intervention, which significantly decreased to two (1%) by the follow-up. All five components of functional limitations showed statistically significant improvement after treatment (P<0.001). Forty-three children (21.5%) were asked about their dental condition by other children before treatment, which decreased to three (1.5%) at follow-up. All six components of emotional and social well-being showed statistically significant improvement after treatment (P <0.001). The perceived outcome results are summarized in Table 4.

**Table 4:** Perceived outcomes after full dental rehabilitation under general anesthesia *A value of P≤0.05 was considered significant.

Outcomes	Before treatment, n (%)	After treatment, n (%)	p-value*
				Oral Symptoms			
Food caught between teeth	43 (21.5)	1 (0.5)	<0.001
Pain in teeth/mouth	191 (95.5)	4 (2)	<0.001
Bad breath	26 (13)	1 (0.5)	<0.001
Mouth sores	17 (8.5)	1 (0.5)	<0.001
Bleeding gums	15 (7.5)	1 (0.5)	<0.001
Functional limitations			
Difficulty chewing coarse food	71 (35.5)	1 (0.5)	<0.001
Difficulty drinking or eating hot/cold foods	37 (18.5)	2 (1)	<0.001
Difficulty eating foods that the child would like to eat	119 (59.5)	1 (0.5)	<0.001
Restricted diet	29 (14.5)	1 (0.5)	<0.001
Troubled sleeping	85 (42.5)	2 (1)	<0.001
Emotional and social well‑being			
Worried about being less attractive	37 (18.5)	1 (0.5)	<0.001
Shy/embarrassed	39 (19.5)	2 (1)	<0.001
Avoided smiling in front of strangers	21 (10.5)	1 (0.5)	<0.001
Did not want to speak or read in class	17 (8.5)	1 (0.5)	<0.001
Asked by other children about the condition	43 (21.5)	3 (1.5)	<0.001
Missed school due to dental causes	35 (17.5)	1 (0.5)	<0.001

Parental satisfaction (Table 5) with treatment was found to be very high after treatment; 97.5% of the parents were satisfied with the overall experience of full-mouth rehabilitation under general anesthesia and 94.5% were willing to let their children be treated under general anesthesia again if the need for repeat treatment were required. Only 7% of the patients said that they would consider multiple visits over general anesthesia if safe sedation were available.

**Table 5:** Parental satisfaction with full mouth rehabilitation under general anesthesia at follow-up.

	Yes, n (%)	No, n (%)
			Overall experience is satisfactory	195 (97.5)	5 (2.5)
Would consider general anesthesia for treatment again if needed	189 (94.5)	11 (5.5)
Would prefer multiple visits over one‑visit general anesthesia if safe sedation was available	14 (7)	186 (93)

Oral health-related quality of life showed statistically significant improvement (P <0.001) after full-mouth rehabilitation under general anesthesia (Table 6). Child Impact Section showed a statistically significant improvement after treatment, with the score decreasing from 15.7±4 to 6.8±1.9 at follow-up. Family Impact Section also showed significant improvement after treatment. The score decreased from 9.6±2.7 to 3.1±2.6 at the follow-up visit. The total ECOHIS decreased from 21.6±9.5 to 9.9±4.2, and a statistically significant difference (P <0.001) was seen with pre- and post-treatment scores.

**Table 6:** ECOHIS scores before and after dental rehabilitation under general anesthesia ECOHIS: Early Childhood Oral Health Impact Scale; SD: standard deviation ^*^A value of P≤0.05 was considered significant

ECOHIS domains	Number of items	Baseline, mean±SD	At follow-up, mean±SD	p-value*
Child impact section	9	15.7±4.1	6.8±1.9	< .001
Child symptoms	1	3.2±0.9	1.1±0.6	< .001
Child function	4	6.7±3.2	2.9±1.2	< .001
Child psychology	2	2.9±1.7	1.5±1.2	< .001
Child self-image and social interaction	2	2.9 ±1.8	1.3±1.2	< .001
Family impact section	4	9.6±2.7	3.1±2.6	< .001
Parental distress	2	4.9±2.3	1.9±1.2	< .001
Family function	2	4.7±1.1	1.2±0.8	< .001
Total ECOHIS score	13	21.6±9.5	9.9 ±4.2	< .001

## Discussion

To provide the best treatment for children with ECC, full mouth rehabilitation under general anesthesia is performed, which involves complete dental rehabilitation in a single visit [9]. The procedure usually lasts a few hours and involves surgical, endodontic, restorative, and preventive dental treatments. Patients are discharged on the same day and kept on recall visits for active surveillance of the treatment as well as new carious lesions [5,8,11].

Pulp therapy for primary teeth was the most common treatment done in the current study. Pulp therapy of primary teeth aims to rehabilitate primary teeth that can be restored to continue their function until they exfoliate to allow permanent successors into their positions [12,13]. The placement of stainless steel crowns on pulp therapy-treated molars is necessary to restore the proper form and contour of the surfaces lost to dental caries. Stainless steel crowns have been used in pediatric dentistry for more than 70 years and are now considered the gold standard for multisurface carious lesions in molars and for teeth after pulp therapy [14]. In the present study, 99% and 96% of patients received restorations and stainless steel crowns, respectively. The placement of restorations in primary teeth should always follow individual caries risk assessment as well as the ability to withstand occlusal forces and return form and function. Because children who are treated under general anesthesia are at high risk for recurrence of new carious lesions, the placement of crowns over restorations should be considered [12]. Of the participants, 13% required extractions. Extractions usually require less time than endodontic procedures and often result in quicker elimination of pain [13]. The use of space maintainers is necessary to maintain the arch space in both the maxilla and mandible until the permanent succedaneous teeth erupt [12]. In the present study, 14% of the children required space maintainers. Parents should be reminded to maintain good oral hygiene for their children as these appliances as well as crowns accumulate plaque and may result in new carious lesions [12,15].

Perceived outcomes refer to the subjective evaluations or assessments that individuals make regarding the results or consequences of a particular action, event, or decision. These assessments are based on their personal perceptions, beliefs, and interpretations of the situation rather than objective or measurable criteria [9]. Perceived outcomes can vary from person to person and can shape an individual's attitudes, behaviors, and decision-making processes [7].

In the perceived outcome questionnaire used in the present study, three domains were evaluated: oral symptoms, functional limitations, and emotional and social well‑being. Pain in the teeth was the most common complaint among the children in the current study. This is in accordance with previously published studies that have found that children usually report to the dentist when pain occurs [3-6]. After treatment, pain was seen only in four patients. This is also in consensus with previously published literature where oral health-related quality of life showed significant improvement after full-mouth rehabilitation [5,16-18]. Forty-three children had complaints of food accumulation before treatment, which decreased to one child after treatment. The restoration of carious teeth with crowns would have resulted in better occlusion and proximal contacts, which in turn reduced the accumulation of food [19]. Fifteen children had bleeding gums when data was collected before treatment, which decreased to one child at the recall visit. This can be attributed to the improvement in oral hygiene instructions after treatment [14].

Significant improvement was seen in the functional outcomes. In the present study, 71 participants had pain while eating coarse food, which decreased to one participant at the follow-up visit. The restoration of occlusion resulting from the placement of crowns helps to improve masticatory function [14,15,20]. The readjustment to normal occlusion occurs within a few weeks, and children can eat without any postoperative problems. Van Der Zee has attributed the readjustment of occlusion to the nature of alveolar bone and jaw growth [21]. Thirty-seven patients had difficulty eating hot and cold food before treatment was initiated, which decreased to two patients at the end of treatment. Comprehensive treatment under general anesthesia results in total dental care, inclusive of preventive measures that play a key role in reducing sensitivity to heat and cold. Parents should play an important role in maintaining oral hygiene practices; otherwise, a relapse of caries can occur [2,8,9]. A total of 119 children complained that they were not able to eat food they liked before the procedure but only one patient had the same complaint at the follow-up visit. A total of 85 participants had trouble sleeping before treatment. Decreased sleeping time and irregular sleep patterns have been found to be risk factors for ECC [22]. After treatment under general anesthesia, only two participants had trouble with sleep. This can be attributed to the adjustment to newly established occlusion after full-mouth rehabilitation, eventually readjusting to a proper occlusion due to the alveolar nature of the bone in children [21]. Twenty-nine children had a restricted diet because of the condition of their teeth before treatment. Only one child had a restricted diet after treatment as parents wanted to keep the diet under control to prevent the relapse of caries as well as the occurrence of ECC in younger siblings.

Emotional and social well-being in children was found to be greatly improved after treatment. Of the 200 participants, 37 were worried about being less attractive before treatment. This could be due to various reasons such as the discoloration of anterior teeth, damaged teeth, or the thought that their smile was not good compared with that of their peers [1,3,14]. Only one child still felt worried about being less attractive at follow-up. Thirty-nine participants were shy before treatment, which decreased to two children at the follow-up. Mathew et al. found that children as young as three years were well aware of self-beauty and were concerned about their looks [14]. Seventeen children did not want to speak or read in class. Of the participants, 43 reported that other children had enquired about their oral conditions, which decreased to three participants after treatment. The unaesthetic appearance of anterior teeth along with the unwillingness to smile and talk are obvious reasons for peers to ask about their teeth [23]. ECC management results in both functional and aesthetic improvement that leads to children asking about the changes seen in their friends [3,5]. Thirty-five participants reported that they missed school for dental causes. This is similar to previous studies, which reported that ECC resulted in loss of school days [3,5,6,12,17,18]. Only one child complained of missing school for dental reasons at the follow-up meeting.

Parental satisfaction plays a key role in pediatric dentistry. Treatment plans are made in discussion with the parents who want the best for their children [14]. Given the aggressive nature of ECC, parents must take time from work to visit the dental clinic as well as other specialists for other underlying conditions [1-4]. Of the parents, 97.5% found the overall experience of full-mouth rehabilitation under general anesthesia satisfactory and 94.5% agreed that they would consider general anesthesia as a means of treatment if it were necessary again. Only 7% said that they would prefer multiple visits over general anesthesia. The overall parental satisfaction was found to be high and similar to that in other studies exploring parental satisfaction after full-mouth rehabilitation of their children under general anesthesia [3,5,6,12,17,18]. A possible reason for few patients preferring multi-visit dental treatment could be minor complications after treatment such as bleeding or painful mastication. Farsi et al. evaluated postoperative complications in children after full-mouth rehabilitation under general anesthesia and found that they were limited to 24 hours after discharge [24]. Postoperative complaints showed significant improvement after 72 hours, and significant improvement in oral health-related quality of life was seen.

In the current study, a significant improvement in oral health was seen after intervention in both the child impact and the family impact sections. The child impact section decreased by nearly half, and the family impact section decreased by almost two-thirds when patients returned for follow-up. In the child impact section, the maximum improvement was seen in child function (6.7±3.2 to 2.9±1.2) and child symptoms (3.2±0.9 to 1.1±0.6). This is similar to results in previously published studies [17,19,24]. The total ECOHIS decreased from 21.6±9.5 to 9.9 ±4.2. This is similar to other studies that have shown significant improvement after full-mouth rehabilitation [6,12,18]. The advantages of a single appointment, fewer complications, and the lack of need for any other behavior modification make full-mouth rehabilitation under general anesthesia an excellent treatment choice for children with ECC [3,5].

Though significant improvement in oral health-related quality of life has been reported after full-mouth rehabilitation for ECC, relapse or recurrence is always a possibility. Maintenance of proper oral hygiene and control of cariogenic diet should be reinforced to parents [4]. Children who have ECC are considered to be at high risk for dental caries in their permanent dentition [1,2]. Hence, the introduction of six-month dental visits can play a key role in developing a dental home as well as starting active surveillance for the follow-up of dental caries [25].

Our study had a few limitations. This prospective interventional cohort study was conducted in a single center in a teaching university dental hospital in India; thus, the results may not be similar to those conducted at other geographical locations. Patients were followed up for a short period, which may not have been enough to evaluate the occurrence of new carious lesions reported with full-mouth rehabilitation after caries. The strengths of the present study were that none of the patients were lost to follow-up. All parents reported back for the follow-up appointment and were able to fill out parental satisfaction, perceived outcome, and oral health-related quality of life surveys.

## Conclusions

The oral health-related quality of life showed significant improvement after full-mouth rehabilitation under general anesthesia. Children were found to have significant relief from pain and could eat and sleep without disturbances after treatment was completed. Pain, dietary restrictions, and concerns about appearance and social interactions significantly diminished following the comprehensive dental treatment provided under general anesthesia. This improvement not only alleviates the suffering of these young children but also contributes to their overall well-being and quality of life.

Parental satisfaction was found to be exceptionally high, with the majority of parents expressing contentment with the treatment process and outcomes. The study underscores the vital role of parental support and cooperation in the management of ECC and highlights the benefits of a single-appointment, comprehensive approach to dental rehabilitation under general anesthesia. While the results of this study provide valuable insights into the perceived outcomes and parental satisfaction associated with ECC treatment, ongoing vigilance, and postoperative care remain essential to prevent relapse and ensure the long-term oral health of these children. Overall, the findings reinforce the importance of early intervention and comprehensive dental care for children with ECC, ultimately improving their oral health and overall quality of life.
